# Efficacy of hydrodilatation in frozen shoulder: a systematic review and meta-analysis

**DOI:** 10.1093/bmb/ldad018

**Published:** 2023-07-26

**Authors:** Daryl Poku, Rifat Hassan, Filippo Migliorini, Nicola Maffulli

**Affiliations:** Faculty of Medicine, University of Southampton, Southampton, SO17 1TW, UK; Faculty of Medicine, University of Southampton, Southampton, SO17 1TW, UK; Department of Orthopaedic, Trauma, and Reconstructive Surgery, RWTH University Hospital, Aachen 52074, Germany; Department of Orthopaedics and Trauma Surgery, Academic Hospital of Bolzano (SABES-ASDAA), Bolzano 39100, Italy; Department of Medicine, Surgery and Dentistry, University of Salerno, Salerno 84084, Italy; Centre for Sports and Exercise Medicine, Barts and The London School of Medicine and Dentistry, Mile End Hospital, London E1 4DG, UK; School of Pharmacy and Biotechnology, Keele University School of Medicine, Thornburrow Drive, Stoke on Trent, Keele ST5 5BG, UK

**Keywords:** frozen shoulder, hydrodilatation, corticosteroid injection

## Abstract

**Introduction:**

It is unclear whether hydrodilatation is beneficial in the management of frozen shoulder compared with other common conservative management modalities. This systematic review evaluates the efficacy of hydrodilatation for the management of frozen shoulder.

**Sources of data:**

A systematic review was conducted in accordance with the Preferred Reporting Items for Systematic Reviews and Meta-Analyses guidelines. An extensive search of PubMed, Embase, Scopus, Cochrane Central, Web of Science and CINAHL databases using multiple keyword combinations of ‘shoulder’, ‘rotator’, ‘adhesive capsulitis’, ‘hydrodilatat^*^’, ‘distension’ since inception of the databases to June 2023 was implemented.

**Areas of agreement:**

Hydrodilatation leads to at least transient more marked improvements in shoulder disability and passive external rotation compared with intra-articular corticosteroid injections.

**Areas of controversy:**

Hydrodilatation improves passive external rotation in the longer term. Moreover, hydrodilatation may be a preferable option over manipulation under anaesthesia, given its lower cost and better patient convenience.

**Growing points:**

Intensive mobilization after hydrodilatation is a promising adjuvant treatment option for patients suffering from a frozen shoulder.

**Areas timely for developing research:**

Although current evidence suggests that hydrodilatation provides a transient improvement in disability in patients with frozen shoulder, its clinical relevance remains unclear. Further research is necessary to establish its role in the management of the condition.

## Introduction

Frozen shoulder, sometimes referred to as adhesive capsulitis, is an insidious musculoskeletal condition that affects the glenohumeral joint. It is characterized by the formation of scar tissue, adhesions and capsular thickening within the shoulder.^1^^,^^2^ Frozen shoulder has a reported prevalence of 2–5% in the general population, rising to 20% in individuals with diabetes mellitus.^3^ Typically, patients present with excruciating pain and reduced passive and active range of motion (ROM) of the glenohumeral joint. Symptoms generally last from 6 months to 2 years. Most patients demonstrate spontaneous resolution of symptoms, and thus conservative management is commonly advised.^4^

Currently, there exists a plethora of conservative management options for patients with frozen shoulder, including analgesia, corticosteroids (oral or intra-articular), physiotherapy, acupuncture, manipulation, suprascapular nerve blockade and hydrodilatation.^5^ First proposed in 1965 by Andren and Lundberg, intra-articular hydrodilatation attempts to expand the joint space through the sheer hydraulic pressure exerted by the injectate.^6^ However, given the marked disability caused by frozen shoulder, some patients may forgo the less invasive hydrodilatation and instead opt for more invasive surgery. This is a possible consequence of the perceived slow nature of symptom improvement with conservative approaches.^7^ Additionally, there remains ambiguity surrounding the effectiveness of hydrodilatation as a treatment method.^8^

Gam et al. compared hydrodilatation to corticosteroid injections alone and identified improvements in shoulder pain and ROM.^9^ However, the results of this study were limited given the high risk of bias. On the contrary, Corbiel et al. and Jacobs et al. found no significant differences when assessing the same treatment modalities.^10^^,^^11^ Furthermore, many studies have examined the efficacy of hydrodilatation amid other treatment options, and thus its specific effects have not always been assessed.^12^

The effectiveness of hydrodilatation may well be short-lived,^10^^,^^11^ as no large study has addressed this particular aspect of the intervention.^10^ Hydrodilatation may potentially lower the prevalence of long-term impairments; however, it remains challenging to determine the number of patients suffering from residual deficiencies.^10^ Most recently, Saltychev et al. demonstrated statistically significant symptomatic improvements with the use of hydrodilatation when assessing its effectiveness in the management of frozen shoulder. However, this effect was deemed not to be clinically relevant.^5^ Thus, amid the incongruent results in the literature, more research is warranted. Nonetheless, hydrodilatation is recommended as part of the patient care pathway co-produced by the British Elbow and Shoulder Society and British Orthopaedic Association.^12^

This review evaluates the current evidence on the efficacy of hydrodilatation for frozen shoulder. This study builds on the previous systematic review by Saltychev et al.,^5^ through the inclusion of recently published randomized controlled trials and prospective and retrospective studies.

## Methods

### Study design

The Preferred Reporting Items for Systematic Reviews and Meta-Analyses (PRISMA) 2020 were used to conduct and report this review.^13^ Our Population, Intervention, Comparison and Outcome framework was as follows:

- Participants: adults, with frozen or painfully stiff shoulders, suffering from discomfort that limits both active and passive glenohumeral joint motions.
- Intervention: glenohumeral joint hydrodilatation (hydrodistension and distension).
- Comparison: intra-articular corticosteroid injections, a placebo, sham, other interventions or no therapy.
- Outcome: all clinically relevant outcomes.
  - Primary: assessment of pain and function or disability.
  - Secondary: ROM, complications and any others.

### Search strategy

Computer searches were conducted on PubMed, Embase, Scopus, Cochrane Central, Web of Science and CINAHL electronic databases from inception to June 18, 2023 for articles assessing hydrodilatation in patients with frozen shoulder. The goal was to increase the search strategy’s sensitivity to increase the likelihood that all relevant studies would be obtained.^14^^,^^15^

Our search clause for the PubMed search was ‘(shoulder OR rotator OR adhesive capsulitis) AND (hydrodilatat^*^ OR distension).’ When conducting searches on the different databases, similar clauses were utilized. We adjusted the search strategy from a previous systematic review^5^ to accommodate our own needs. The search was restricted to humans only, and the reference management software EndNote was used to organize its results. The relevance of the cited studies’ references was also examined. A step-by-step process, which involved team meetings to improve the search strategy and settle disagreements, was utilized to ensure that the searches were producing relevant studies.^16^

### Study screening

All references were downloaded from the Rayyan reference management software, and duplicates were removed before screening the title and abstract. The full texts of the remaining articles were examined after two authors (DP and RH) independently assessed the titles and abstracts. A consensus meeting between the two authors was organized to settle disputes that arose during research screening and selection. If no consensus could be reached, the senior author (NM) was contacted for a final decision.

### Study selection

Only peer-reviewed journals were considered. There were limited restrictions on the study design within the selection criteria, which increased the likelihood of identifying pertinent studies. Thus, randomized controlled trials, prospective and retrospective comparative studies and case series were included. Level I–IV studies, according to the Oxford Centre for Evidence Based Medicine, were identified and included in our analysis. The hydrodilatation technique and follow-up period had to be well described in all included studies, which had to use at least one validated clinical outcome score or assess change in ROM. Studies needed to be published in English, and had to have recruited at least 10 adult participants. Exclusion criteria were reviews, case reports, experiments on animals, cadavers or *in vitro* and letters to editors. We also excluded articles with no information on hydrodilatation intervention, diagnosis, follow-up, clinical examination and statistical analysis.

To prevent bias, all authors read, evaluated and discussed the included and excluded studies and the relative list of references. The senior investigator (NM) made the final decision if there was a disagreement among the investigators on the inclusion and exclusion criteria.

### Data extraction

Data extracted from each study included the following: author name, study year; study design (level of evidence); number of patients (shoulders); mean age (range) (years); diabetes mellitus diagnosis; Coleman Methodology Score (CMS); imaging assessment; duration of symptoms (average) (months); outcome measures (time intervals); regimen and modification of the distension arms; comparative intervention arm; hydrodilatation technique; and complications. Data were entered in a custom Excel spreadsheet by all the investigators independently. A standardized form, based on the recommendations of the Cochrane Handbook for Systematic Reviews of Interventions Version 5.1.0, Chapter 7, was used for data extraction for the meta-analysis.^17^ Discussions with the senior author (NM) allowed the resolution of any discrepancies.

### Quality assessment

The methodological quality was assessed according to the CMS.^18^ Modifications of the CMS were made to make it pertinent for the systematic review of frozen shoulder (Table 1). Each study was scored by two reviewers (DP and RH) independently and in duplicate for each of the criteria adopted to give a total CMS between 0 and 100. A study design that eliminates the impact of chance, bias and confounding variables would receive a score of 100. Disagreements were resolved by discussion. The CMS is divided into sections, each of which is based on a component of the CONSORT statement (for randomized controlled trials) with modifications to accommodate various study designs.

**Table 1:** Modified Coleman Methodology Score

Part A: Only one score to be given for each of the seven sections		
**Study**—**number of patients**	< 30	0
	30–50	4
	51–100	7
	>100	10
**Mean follow-up**	< 6 months	0
	6–12 months	4
	12–18 months	7
	>18 months	10
**Injection approach**	Different approaches were used and outcomes were not reported separately	0
	Different approaches were used and outcomes were reported separately	7
	Single approached	10
**Type of study**	Retrospective cohort study	0
	Prospective cohort study	10
	Randomized controlled trial	15
**Description of diagnosis**	Described without % specified	0
	Described with % specified	5
**Descriptions of injection technique**	Inadequate (not stated, unclear)	0
	Fair (technique only stated)	5
	Adequate (technique stated, details of surgical procedure given)	10
**Description of post-injection rehabilitation**	Described	5
	Not described	0
**Part B: Scores may be given for each option in each of the three sections if applicable**		
**Outcome criteria**	Outcome measures clearly defined	2
	Timing of outcome assessment clearly stated	2
	Use of outcome criteria that have reported reliability	3
	General health measures included	3
**Procedure of assessing outcomes**	Subjects recruited	5
	Investigator independent of injection clinician	4
	Written assessment	3
	Completion of assessment by patients themselves with minimal investigator assistance	3
**Description of subject selection process**	Selection criteria reported and unbiased	5
	Recruitment rate reported	
	>90%	5
	<90%	0

### Statistical analysis

The meta-analysis was performed using Review Manager, version 5.4 (The Cochrane Collaboration). The *I*^2^ statistic was used to test for statistical heterogeneity and was assessed as follows: 0% < *I*^2^ < 25%, low heterogeneity; 25% < *I*^2^ < 50%, moderate heterogeneity; and *I*^2^ > 50%, high heterogeneity.^18^ This effectively describes the percentage of variation across studies originating more from heterogeneity than from chance. We used the random-effects model because outcome measurements were taken at different time points, and the different phases of frozen shoulder increases the risk of heterogeneity. Data for quantitative analysis were extracted at two-time points: the first follow-up post-intervention and the last follow-up post-intervention. The Egger’s test and a funnel plot were used to evaluate the publication bias.

When just the interquartile range (IQR) was provided, IQR/1.35 was used to calculate the standard deviation (SD). According to the Cochrane Handbook for Systematic Review of Interventions Version 5.1.0, Chapter 7, the mean was presumed to be the same as the median when only the median was given.^17^ SD was computed as (max-min)/4 when only the range was given. Cohen’s *d*—a standardized mean difference (SMD) in variable change between groups—was used to calculate the effect sizes.

Variables were measured by the SMD with 95% confidence intervals (95% Cis). Data synthesis was initiated for each included study by combining pertinent reported outcomes stratified by pain, disability and ROM at pre-determined time points (earliest and latest follow-ups).

In all analyses, a *P*-value < 0.05 was considered statistically significant. Sensitivity analysis was conducted to evaluate the reliability of the effects. One study was eliminated at that time, and studies with very heterogeneous findings were also eliminated.

## Results

### Study identification and selection

**Fig. 1:**
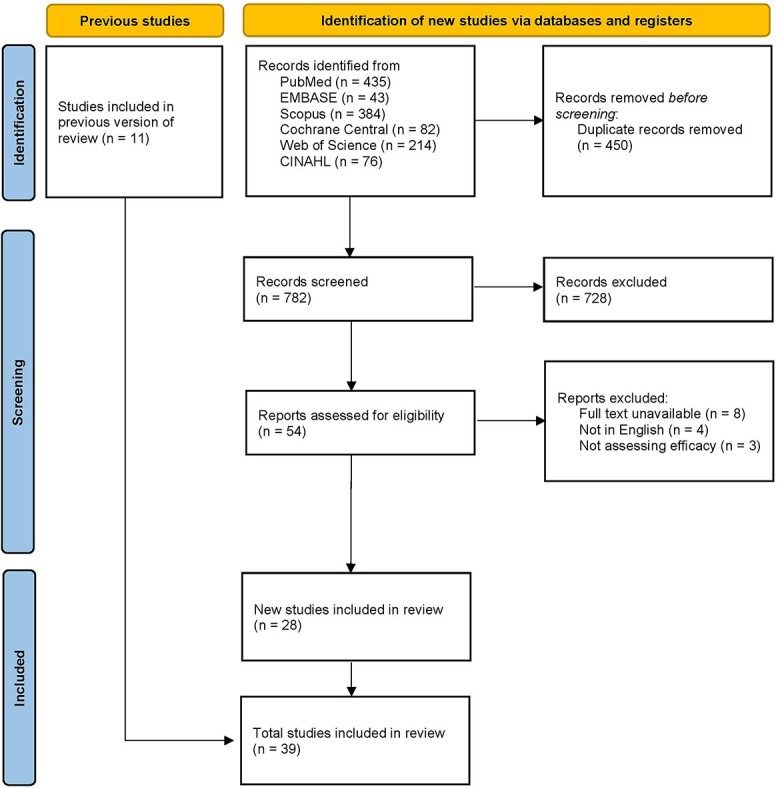
Prisma flow diagram.

Our initial search yielded 1234 articles, with a total of 452 left following the removal of duplicates. We then screened the titles and abstracts of the remaining articles and retained 54 articles for full-text evaluation, which resulted in 39 studies (Fig. 1).

### Demographics

A total of 2623 participants and 2632 shoulders were included. The number of participants recruited in each study varied from 22 to 250. Data on the incidence of diabetes were reported in 16 included studies. Of the 1187 patients, 224 (18%) were diabetic (Table 2).

**Table 2:** Details of included articles

Authors	Study design (level of evidence)	No. of patient (shoulders)	Mean age (range) (years)	Diabetes mellitus diagnosis	CMS
Dai et al. [2022]^19^	Prospective randomized controlled trial (II)	66	ACR + HD group: 53.3 HD group: 52.8		77
Wang et al. [2022]^20^	Prospective, double-blind, randomized controlled trial (I)	84	HD group 1: 54.3 (52.0–56.67) HD group 2: 55.1 (52.6–57.6)	11	71
Albana et al. [2022]^21^	Retrospective cohort study (IV)	31	HD group: 55.9 HD + SSN group: 52.8		47
Debeer et al. [2021]^22^	Prospective case series (IV)	72	53 (38–70)	9	63
Saoji et al. [2021]^23^	Prospective cohort study (IV)	40			55
Wang et al. [2021]^24^	Prospective randomized control trial (II)	64	Posterior glenohumeral recess group: 54.0 (51.4–56.5) Rotator cuff interval group: 52.4 (50.1–54.7)	12	76
Makki et al. [2021]^25^	Retrospective case series (IV)	250	59 (21–73)	27	36
Paruthikunnan et al. [2020]^26^	Prospective double-blinded randomized control trial (I)	88	Distension group: 56.5 (40–77) Non-distension group: 54.9 (39–68)	46	78
Rae et al. [2020]^27^	Single-arm retrospective observational design (IV)	90	58		56
Ainen et al. [2020]^28^	Retrospective case series (IV)	39	54.3	8	43
Saoji et al. [2020]^29^	Prospective cohort study (IV)	40			58
Baig et al. [2019]^30^	Prospective case series (IV)	150	43.0 (18–60)		51
Kim et al. [2019]^31^	Case Controlled, Retrospective, Comparative Study (IV)	47	Non-pumping group: 55.4 Pumping group: 56.5		56
Gallacher et al. [2018]^32^	Randomized prospective controlled trial (II)	50	HD group: 55.2 (50.6–59.8) ACR group: 52.6 (48.7–56.5)	8	79
Haughton et al. [2018]^33^	Retrospective case series (IV)	76	55.5 (43–66)	11	58
Sinha et al. [2017]^34^	Prospective case series (IV)	118	52.6 (28–73)	26	70
Lee et al. [2016]^35^	Prospective randomized controlled trial (II)	64	HD group: 55.9 IAS group: 53.8		70
Sharma et al. [2016]^36^	Prospective randomized controlled trial (II)	106	HD group: 53 IAS group: 52 TAU group: 54		72
Yoon et al. [2016]^37^	Prospective randomized controlled trial (I)	86	HD group: 54.0 IAS group: 53.0 SAI group: 57.0	12	77
Yoong et al. (2015)^38^	Prospective case series (IV)	22	55.0 (32–71)	3	52
Afsar et al. (2015)^39^	Prospective case series (IV)	63	51.9 (44–73)		63
Ahn et al. [2015]^40^	Prospective cohort study (IV)	121	HD group: 54.6 IAS group: 55.2		57
Lee et al. [2015]^41^	Prospective randomized controlled trial (I)	64	Hypertonic saline group: 56.5 Normal saline group: 53.7		60
Park et al. [2014]^42^	Randomized controlled trial (II)	53	56		47
Bae et al. [2014]^43^	Randomized controlled trial (II)	54	Fluoroscopy-guided HD group: 53.3 Ultrasonography-guided HD group: 53.9		62
Clement et al. [2013]^44^	Prospective cohort study (IV)	51 (53)	52 (34–75)	12	77
Park et al. [2013]^45^	Prospective randomized controlled trial (II)	90	HD group: 55.2 IAS group: 56.3		79
Park et al. [2012]^46^	Prospective randomized controlled trial (II)	48	Sono-guided HD group: 56.0 Fluoroscopically guided HD group: 56.4		66
Ng et al. [2012]^47^	Prospective randomized trial (II)	28 (30)	HD group: 53 (44–69) MUA group: 52 (44–61)	9	63
Trehan et al. [2010]^48^	Prospective case series (IV)	36	54.6 (26–74)		74
Jacobs et al. [2009]^49^	Prospective randomized controlled trial (I)	53	HD group: 57.0 MUA group: 56.5		71
Tveitå et al. [2008]^50^	Randomized controlled trial (II)	76	51.5		60
Quraishi et al. [2007]^51^	Prospective randomized controlled trial (II)	36 (38)	55.2 (44–70)	6	61
Watson et al. [2007]^52^	Prospective clinical series (IV)	53	54.7		76
Buchbinder et al. [2004]^53^	Double blind, randomized, placebo-controlled trial (I)	48	HD group: 57.2 Placebo group: 57.5	13	67
Vad et al. [2003]^54^	Prospective case series (IV)	22	41.3 (29–54)		76
Gam et al. [1998]^9^	Randomized controlled trial (II)	22	53 (40–65)		66
Jacobs et al. [1991]^55^	Prospective randomized controlled trial (II)	47 (50)	HD group: 55 Distension only group: 53 IAS group: 52		52
Hsu et al. [1991]^56^	Prospective cohort study (IV)	75	Women: 54 (41–73) Men: 49 (38–61)	11	48

HD = hydrodilatation; SSN = supracapsular nerve block; ACR = arthroscopic capsular release; IAS = intra-articular steroid; SAI = subacromial injection; MUA = manipulation under anaesthesia; TAU = treatment as usual

### Study identification and selection

A total of 20 studies (51.3%) used imaging, such as ultrasound or magnetic resonance imaging, to confirm the diagnosis of frozen shoulder. The hydrodilatation procedures were performed under ultrasound or fluoroscopic guidance. In 21 studies, the hydrodilatation was administered through the posterior approach, in 13 through the anterior approach, and in 1 using both anterior and posterior approaches. Inclusion and exclusion criteria were overall quite similar across most articles. The volume of mixture injected for hydrodilatation ranged from 9 to 100 mL. Typically, the hydrodilatation mixture consisted of corticosteroids, local anaesthetic and normal saline solution, and only one study used a combination of hyaluronic acid and lidocaine.^45^ Intra-articular corticosteroid injections were the most commonly utilized reference therapy. Arthroscopic capsular release (ACR), manipulation under anaesthesia (MUA), placebo (arthrogram), general physical therapy and treatment as usual (i.e. physical therapy and oral medication) were also used (Table 3).

**Table 3:** Frozen shoulder management

Authors	Imaging assessment	Duration of symptoms (average) (months)	Outcome measures (time intervals)	Regimen and modification of the distension arms	Comparative intervention arm	Hydrodilatation technique	Complications
Dai et al. [2022]^19^	RX, MRI	ACR + HD group: 5.4 HD group: 4.5	Passive ROM, VAS (baseline, 1, 4, 12, 24 weeks, 1 year) UCLA, DASH (baseline, 12 weeks, 24 weeks, 1 year)	**ACR + HD group:** 50 mg triamcinolone acetonide, 100 mg ropivacaine with saline to a volume of 20 mL, plus ACR **HD group:** 50 mg Triamcinolone acetonide, 100 mg ropivacaine with saline to a volume of 20 mL		Posterior approach with landmark guidance	There were no complications such as infection, nerve or vascular injury.
Wang et al. [2022]^20^	RX, ultrasound, MRI	HD group 1: 6.0 HD group 2: 5.6	SPADI, VAS, ROM (baseline, 6, 12 weeks)	**HD group 1:** 4 mL of 40 mg triamcinolone acetonide, 4 mL of 2% lidocaine, 12 mL of normal saline **HD group 2:** 1 mL of 10 mg triamcinolone acetonide, 4 mL of 2% lidocaine, 15 mL of normal saline		Posterior approach with ultrasound guidance	No adverse events were reported in either group.
Albana et al. [2022]^21^	RX		VAS (intra-injection, 1, 6 months) ASES, DASH (1, 6 months)	**HD group**: 15–20 mL of saline, 40 mg of triamcinolone **SSNB + HD group**: 15–20 mL of saline, 40 mg of triamcinolone plus suprascapular nerve block		Posterior approach with ultrasound guidance	
Debeer et al. [2021]^22^	RX, ultrasound, MRI	8	SPADI, HADS, TSK-11, VAS (baseline, 3 months) Constant-Murley Score (baseline, immediately post-HD, 3 months)	**HD group:** 1 mL of 40 mg of methylprednisolonacetate, 15 mL of 0.5% marcaine, 20 mL of normal saline		Fluoroscopic guidance	
Saoji et al. [2021]^23^	RX, ultrasound		VAS (baseline, 3 weeks)	**HD with steroid group:** Up to 16 mL of normal saline, 2 mL Bupivacaine hydrochloride, 2 mL of 80 mg Methylprednisolone **HD without steroid group:** 18 mL of sterile normal saline, 2 mL Bupivacaine hydrochloride (5 mg/ml)		Fluoroscopic or ultrasound guidance	
Wang et al. [2021]^24^	RX, ultrasound	Posterior glenohumeral recess group: 6.7 Rotator cuff interval group: 6.1	SPADI, VAS, ROM (baseline, 6, 12 weeks)	**Posterior glenohumeral recess group**: 4 mL of 40 mg triamcinolone acetonide, 4 mL of 2% lidocaine, 12 mL of normal saline **Rotator cuff interval group:** 4 mL of 40 mg triamcinolone acetonide, 4 mL of 2% lidocaine, 12 mL of normal saline		Posterior approach with ultrasound guidance	The procedure was well tolerated by the patients in both approaches, and no serious adverse events were observed. Two patients (one in each group) reported significant post-injection pain (VAS score > 4) on the first day after the intervention, which resolved spontaneously without the need for additional treatments.
Makki et al. [2021]^25^			ROM (baseline, 3 months, 12 months) Pain scores (baseline, 3 months)	**HD group:** 1 mL of 40 mg Kenalog, 10 mL of 0.25% levobupivacaine, 20 mL of 0.9% NaCl solution		Anterior approach with fluoroscopic guidance	
Paruthikunnan et al. [2020]^26^	RX	Distension group: 5.0 Non-distension group: 5.1	SPADI (baseline, 1.5, 3, 6 months)	**IAS + HD (Distension**) **group**: 12–18 mL of 0.25% bupivacaine, 2 mL of 80 mg methylprednisolone acetate	**IAS (Non-distension) group**: 2 mL of 0.25% bupivacaine, 2 mL (80 mg) of methylprednisolone acetate	Posterior approach with ultrasound guidance	Other than intra-procedural pain, there were no other significant complications. The average VAS pain score for the non-distension group was 7.4(SD: 1.1) while that in the distension group was significantly higher—8.6(SD:1.0); p < 0.001. 4 patients from the distension group and 3 from the non-distension group had very severe post-injection pain on the night following the intervention, which were controlled by analgesics. None of the patients had any features of septic arthritis post-intervention.
Rae et al. [2020]^27^	RX		SPADI, UEFI, VAS, EQ-5D-5 L (baseline, 6, 12, 24 weeks) ROM (baseline, 24 hours post-HD)	**HD group:** 40 mg of triamcinolone acetonide, 4 mL of 1% lidocaine, 25 mL of 0.9% sodium chloride		Posterior approach with ultrasound guidance	None
Ainen et al. [2020]^28^	Ultrasound	10.2	NRS, ROM (baseline, 6 weeks)	**HD group:** 40 mL of normal saline, 40 mg of kenalog, 2 mL of 0.25% marcaine		Posterior approach with ultrasound guidance	Procedure had to be abandoned in one case because of pain
Saoji et al. [2020]^29^	RX, ultrasound		SPADI (baseline, 3, 6, 12, 16 weeks)	**HD with steroid group**: Up to 16 mL of sterile normal saline, 2 mL of Bupivacaine hydrochloride (5 mg/ml) and 2 mL Depomedrol (80 mg Methyl Prednisolone) **HD without steroid group**: 18 mL sterile normal saline, 2 mL of Bupivacaine hydrochloride (5 mg/ml)		Anterior approach with either fluoroscopic or ultrasound guidance	
Baig et al. [2019]^30^	RX		VAS, OSS (baseline, 1 month)	**HD group:** 10 mL of 80 mg of triamcinolone, lidocaine 1%, 40 mL of warm saline			
Kim et al. [2019]^31^	RX, ultrasound, MRI	Nonpumping group: 6.1 Pumping group: 5.6	Passive ROM, VAS, SPADI (baseline, 3, 6 months)	**HD (Non-pumping) group**: 1 mL of 40 mg Triamcinolone, 10 mL of 1% lidocaine, 19 mL of normal saline solution **HD (Pumping) group**: 1 mL of 40 mg Triamcinolone, 10 mL of 1% lidocaine, 19 mL of normal saline solution		Posterior approach with ultrasound guidance	No serious complications such as loss of sensation, motor control in the affected arm, infection, or symptoms attributable to side effects of hydraulic distension. Some patients reported some side effects such as facial flushing (total *n* = 4: the pumping group, *n* = 2; the nonpumping group, *n* = 2), local depigmentation of the skin (the pumping group, *n* = 1) and disturbance of the menstrual pattern (total *n* = 5: the pumping group, *n* = 2; the nonpumping group, *n* = 3).
Gallacher et al. [2018]^32^	RX, ultrasound		OSS, EQ-5D VAS, Passive ROM (baseline, 6 weeks, 3 month, 6 months)	**HD group:** 1 mL of 80 mg Triamcinolone, 4 mL of 2% lidocaine, 40 mL of normal saline	**ACR group**: Arthroscopic capsular release through a rotator interval portal to the 5 o’clock position.	Anterior approach with fluoroscopic guidance	No complications were noted in either group.
Haughton et al. [2018]^33^			OSS (baseline, post-intervention)	**HD group:** 40 mg of Kenalog, 10 mL of 0.5% chirocaine, saline and contrast		Fluoroscopic guidance	There were no complications observed in any patient during the HD process.
Sinha et al. [2017]^34^	RX, ultrasound		QDASH, OSS (4 weeks, 3, 6, 12 months)	**HD group:** 8–10 mL of 1% lignocaine, 1 mL of 80 mg triamcinolone, 15 mL of 0.25% bupivacaine, 40–60 mL of normal saline		Posterior approach with ultrasound guidance	The procedure was well tolerated by all patients. The patient group experienced few adverse events/complications of the procedure. One patient developed transient suprascapular nerve palsy because of leakage of anaesthetic into the supraglenoid notch region. Patients often feel some discomfort and sometimes feel dizzy in the first few minutes of/after the procedure. Patients were advised to be accompanied for the procedure and not to drive for the rest of the day.
Lee et al. [2016]^35^	RX, ultrasound, MRI	HD group: 8.2 IAS group: 7.8	SPADI, VAS, Passive ROM (baseline, 3, 6, 12 weeks)	**HD group**: Normal saline, 1 mL of 40 mg/mL triamcinolone acetonide, 6 mL of 1% lidocaine	**IAS group**: 1 mL of 40 mg/mL triamcinolone acetonide, 3 mL of 1% lidocaine	Posterior approach with ultrasound guidance	There were no serious complications (eg, infection) other than facial flushing on days 2 to 5 after injection (1 participant in the IACI group and 2 participants in the capsule-preserved hydrodilatation with corticosteroid group) and dizziness because of vasovagal reaction during the injection (3 participants in the capsule-preserved hydrodilatation with corticosteroid group).
Sharma et al. [2016]^36^		HD group: 7.0 IAS group: 7.5 TAU group: 6.0	SPADI (baseline, 4, 8 weeks, 1 year) NPRS, Passive ROM (baseline, 4, 8 weeks)	**HD group**: 1 mL of Triamcinolone 20 mg, 3 mL Lidocaine, 8 mL to 20 mL of sodium chloride	**IAS group**: 1 mL of 20 mg Triamcinolone, 3 mL of lidocaine **TAU group**: physiotherapy and oral pain medication	Posterior approach with landmark guidance	Six patients (17%) in the IS group and four (12%) patients in the ISD group experienced minor transitory side-effects such as flushing and after-pain. No incidences of other side effects were reported.
Yoon et al. [2016]^37^	RX, ultrasound	HD group: 9 IAS group: 9 SAI group: 9	VAS, SST, Constant score, Passive ROM (baseline, 1, 3, 6 months)	**HD group**: 1 mL of 40 mg triamcinolone, 4 mL 2% lidocaine and 40 mL normal saline	**IAS group**: 1 mL of 40 mg triamcinolone, 4 mL of 2% lidocaine and 5 mL of normal saline **SAI group**: 1 mL of 40 mg triamcinolone, 4 mL of 2% lidocaine and 5 mL of normal saline	**IAS & SAI group**: Anterior approach with ultrasound guidance **HD group**: Anterior approach with fluoroscopic guidance	Two patients in the IAI group and 1 patient in the SAI group compained of temporary mild dizziness and nausea after the injection. One patient in the HD group reported transient loss of sensation and motor control in the injected arm for a few hours after the injection, but these symptoms recovered with sequelae. One other patient in the HD group reported transient hypotensive syncope immediately after the injection, but the patient fully recovered after several minutes.
Yoong et al. (2015)^38^	Ultrasound		VAS (baseline, immediately after injection, during the first 24 hrs, during second 24 hrs, 2 weeks) Symptom improvement (4 months) OSS (baseline, 4 months)	**HD group**: 20 mL of a mixture of 1% lidocaine and 0.5% bupivacaine (50:50 mixture)		Anterior approach with ultrasound guidance	Rotator interval hydrodilatation was well tolerated in all patients with no immediate complications
Afsar et al. (2015)^39^	RX		Simple shoulder test, SST (baseline, 6 months) VAS (baseline, every 3 weeks for 6 months post injection)	**HD group**: 3 mL of 1% lidocaine and 40 mL of normal saline		Anterior approach	
Ahn et al. [2015]^40^	RX, ultrasound	HD group: 7.4 IAS group: 7.2	SPADI, VNS, passive ROM (baseline, 1, 3, 6 months)	**HD group**: 19 ml of 0.5% lidocaine plus 1 mL of 30 mg ketorolac	**IAS group**: 4 mL of 0.5% lidocaine plus 1 mL of 40 mg triamcinolone	Posterior approach with ultrasound guidance	Two patients in US guided IA steroid injection group complained pain due to steroid-induced synovitis which resolved spontaneously within 1 week. Patients in US-guided IA ketorolac injection with capsular distension group reported two cases of dizziness, transient muscle weakness after injection. All reported symptoms had resolved at discharge after monitoring of the symptoms in the recovery room. There were no severe complications, such as septic arthritis, allergic reactions, dyspepsia, bleeding, gastric ulceration and renal dysfunction.
Lee et al. [2015]^41^	RX, Ultrasound	HD (hypertonic saline) group: 4.8 HD (normal saline) group: 6.5	SPADI, passive ROM (baseline, 2 weeks)	**HD (hypertonic saline) group**: 4 mL of 1% lidocaine and 1 mL of 10 mg triamcinolone with 3% NaCl **HD (normal saline) group**: 4 mL of 1% lidocaine and 1 mL of 10 mg triamcinolone with 0.9% NaCl		Posterior approach with ultrasound guidance	No side effects of hypertonic saline injection, including great soreness, pain and other severe conditions, were reported during the study. However, there was mild discomfort resulting from the needle injection and capsule distention in both groups.
Park, et al. [2014]^42^			VNS, SPADI, Constant score (baseline, 4 weeks after final treatment)	**HD + IM group**: 1 mL of 40 mg of triamcinolone, 3 mL of 1% lidocaine and 10 mL of normal saline **HD group**: 1 mL of 40 mg of triamcinolone, 3 mL of 1% lidocaine and 10 mL of normal saline	**IM group**: intensive mobilization without injection **GPT group**: general physical therapy without injection	Anterior approach with fluoroscopic guidance	
Bae et al. [2014]^43^		Fluoroscopy-guided HD group: 7.2 Ultrasonography-guided HD group: 6.9	SPADI, VNS, passive ROM, hand grip, pinch power (baseline, 1 week, 5 weeks, 9 weeks)	**HD (Fluoroscopy-guided) group**: 2% lidocaine (5 mL), contrast dye (5 mL), triamcinolone (40 mg) and normal saline (9 mL), in a total fluid volume of 20 mL **HD (Ultrasonography-guided) group**: 2% lidocaine (5 mL), triamcinolone (40 mg) and normal saline (14 mL), for a total of 20 mL fluid volume		Anterior approach with fluoroscopic guidance and posterior approach ultrasound guidance	
Clement et al. [2013]^44^			OSS, VAS (baseline, 2 days, 1 month, 14 months) ROM (baseline, 2 days, 1 month)	**HD**: 10 mL of 1% lidocaine and 40 mg (diabetic patients) or 80 mg (non-diabetic patients) of Kenalog (triamcinolone), up to 40 mL of warmed saline		Fluoroscopic guidance	One patient developed septic arthritis after the distension procedure. This required an emergent arthroscopic washout and an extended course of antibiotics. One distension procedure was aborted because the patient was unable to tolerate the procedure due to pain. Both patients were included in the final analysis. No other complications or adverse effects were reported.
Park et al. [2013]^45^	RX, ultrasound	HD group: 5.3 IAS group: 5.3	SPADI (baseline, 2 weeks, 6 weeks) VNS (baseline, 2 weeks and 12 weeks) Passive ROM (baseline, 2 weeks, 6 weeks)	**HD group:** 18 mL of 0.5% lidocaine plus 2 mL of sodium hyaluronate 20 mg plus capsular distension	**IAS group**: 4 mL of 0.5% lidocaine plus 1 mL of 40 mg triamcinolone	Posterior approach with ultrasound guidance	Two patients in group A and 1 patient in group B experienced pain because of needle contact to the labrum, and 12 patients in group B reported pain during capsular distension. No severe complications, such as vasovagal syncope, allergic reactions, steroid-associated infection, adipose tissue atrophy, or toxic reactions, including dizziness, were observed after injections.
Park et al. [2012]^46^	RX, Ultrasound	Sono-guided HD group: 7.7 Fluoroscopically guided HD group: 7.4	VNS, SPADI and ROM (baseline, 2 weeks and 6 weeks)	**HD group:** 10 cc non-ionic contrast media and 0.5% of lidocaine 10 cc containing 20 mg triamcinolone (total 20 cc)		Posterior approach with fluoroscopic and ultrasound guidance	Hot flush, as a side effect, was seen in 2 patients from each group, with no statistical differences between the two groups; no other side effects were reported.
Ng et al. [2012]^47^	RX	HD group: 17 MUA group: 17	ROM, DASH, VAS (baseline, 6 weeks, 6 months)	**HD group**: 4 mL of 1% lidocaine, 4 mL of 0.5% bupivacaine, 80 mg of Depo-Medrone plus 20 mL of air	**MUA group**: Manipulation under anaesthesia followed by an intra-articular injection of 4 mL of 1% lidocaine, 4 mL of 0.5% bupivacaine and 2 mL of 80 mg Depo-Medrone	Anterior approach with fluoroscopic guidance	There were no complications encountered during either treatment in the trial. In particular, there were no neurovascular injuries, glenohumeral dislocations or fractures. Two patients who underwent capsular distension continued to have persistent pain and restricted ROM of their shoulders. They were offered MUA after the 6 months follow-up.
Trehan et al. [2010]^48^	RX, MRI, Ultrasound		SDQ-UK, OSS	**HD group**: 10 mL of 0.5% bupivacaine, 2 mL of 80 mg triamcinolone and > 25 mL of normal saline		Posterior approach with fluoroscopic guidance	There were no reported adverse incidents reported.
Jacobs et al. [2009]^49^	RX		Constant score, VAS score (baseline, 2, 6 and 12 weeks, and then at 6, 9, 12, 18 and 24 months) SF-36 (baseline, 24 months)	**HD group**: 1 mL of 40 mg triamcinolone, 5 mL of 2% lignocaine, 10 mL of 0.25% bupivacaine and 5 mL of air	**MUA group:** Manipulation under anaesthesia	Posterior approach	No systemic or local complications were noted from either treatment modality.
Tveitå et al. [2008]^50^	RX	HD group: 7 IAS group: 7	SPADI, Active and Passive ROM (baseline, 6 weeks)	**HD group**: 4 mL contrast medium, 2 mL triamcinolone acetonide, 4 mL local anesthetic and 10 mL saline	**IAS group**: 3–4 mL contrast medium, 2 mL triamcinolone acetonide and 3–4 mL of bupivacaine hydrochloride	Anterior approach with fluoroscopic guidance	Patients recorded pain intensity related to the injection procedures. Most patients reported ‘no pain’ or ‘discomfort’ when describing the procedure. However, six patients in the INJ group and five patients in the DIL group felt that the injections were very painful. Other possible side effects were reported by 20 patients in the INJ group and 14 patients in the DIL group. These were usually mild and lasted only for a few days. Most frequent were complaints over flushing or disturbances in heat regulation (INJ group *n* = 13, DIL group *n* = 9). Two patients in each group reported a minor loss of sensation and motor control in their affected arm. Some patients complained over loss of sleep, nausea or dizziness. One patient in the DIL group developed a glenohumeral joint infection which was identified 5 days after the last injection. He immediately underwent arthroscopic surgery and was treated with infusions of cloxacillin for two weeks, with a good result. His baseline SPADI score was 38, and the score at follow-up was 50. One patient in the INJ group developed breast cancer during the study period.
Quraishi et al. [2007]^51^	RX	HD group: 37.4 weeks MUA group: 39.8 weeks	VAS, Constant score, ROM (baseline, 2 months, 6 months) Satisfaction levels (final follow up)	**HD group**: 10-55 mL normal saline	**MUA group**: Manipulation under anaesthesia permitting restoration of shoulder movement	Anterior approach with fluoroscopic guidance	
Watson et al. [2007]^52^	RX, ultrasound	Primary contracture group: 6.5 Secondary contracture group: 9	SPADI, Shoulder Disability Index (SDI), self-rating of function, active ROM (baseline, 3 days, 1 week, 3 months, 1 year, 2 years)	**HD group:** 1 mL of triamcinolone acetonide, 10 mL bupivacaine 0.5%, sterile sodium chloride 0.9%		Anterior approach with fluoroscopic guidance	
Buchbinder et al. [2004]^53^	RX	HD group: 118 days Placebo group: 114 days	SPADI, Problem Elicitation Technique (PET), Pain vertical Likert scale, ROM (baseline, 3 weeks, 6 weeks, 12 weeks)	**HD group**: 1 mL of 40 mg methylprednisolone acetate and up to 82 mL normal saline (total 30–90 mL)	**Placebo group**: 6 mL of contrast media	Anterior approach with fluoroscopic guidance	No serious adverse effects were reported by participants in either group, but more participants in the active group had pain at the time of the procedure or lasting up to 48 hours than in the placebo group
Vad et al. [2003]^54^	MRI		L’lnsalata Shoulder Rating Questionnaire—LSRQ (baseline, 3 weeks, minimum 1 year) Hannafin ROM protocol (baseline, immediately post-HD, 3 weeks, minimum 1 year)	**HD group**: 15 mL of 1% lidocaine with normal saline (range 40–100 mL)		Posterior approach with fluoroscopic guidance	
Gam et al. [1998]^9^	RX	HD group: 5 IAS group: 4.5	Severity of disorder, passive ROM, VAS, daily usage of analgesia, type and number of side-effects (baseline, 3 weeks, 6 weeks, 12 weeks)	**HD group**: 20 mg of triamcinolonhexacetonid and 19 mL of 0.5% lidocaine	**IAS group**: 20 mg of triamcinolonhexacetonid	Posterior approach with ultrasound guidance	No other side-effects were reported except the two cases of unacceptabale pain after injection
Jacobs et al. [1991]^55^	RX	HD group: 8 Distension only group: 6 IAS group: 6	Analgesic use, severity of pain in relation to daily activities, severity of pain with resisted shoulder movement, active and passive ROM (baseline, 6, 12, 16 weeks) Shoulder dynanometry (2 week intervals for 16 weeks)	**Distension only group**: 6 mL of 0.25% bupivacaine and 3 mL air (total 9 mL) **HD group**: 1 mL of 40 mg triamcinolone acetonide, 6 mL of 0.25% bupivacaine and 3 mL air (total 10 mL)	**IAS group**: 1 mL of 40 mg triamcinolone acetonide (total 1 mL)	Posterior approach with landmark guidance	No patient developed intra-articular sepsis or a supra scapular nerve palsy after the intra-articular injections.Two patients, however, developed temporary (<24 hours) facial flushing after their steroid injection.
Hsu et al. [1991]^56^		7.6	Percentage of pain relief, range of flexion and abduction, functional score, strength of abduction (baseline, 1, 2, 3, 5, 8, 12 weeks)	**HD + physiotherapy group**: normal saline 100-150 mL with standardized physiotherapy	**MUA group**: manipulation under anaesthesia and standard physiotherapy **Physiotherapy group**: standardized physiotherapy with short wave diathermy, interferential therapy and proprioceptive neuromuscular facilitation	Posterior approach	There were no significant complications. Superficial wound infection, which settled with eusol dressings, occurred in two cases in group D.

### Outcomes measurements

The included studies used several outcome measures. The visual analogue score (VAS) was used in 21 articles; the Shoulder and Pain Disability Index (SPADI) in 18 studies; the Oxford Shoulder Score (OSS) in seven studies; the Disabilities of the Arm, Shoulder and Hand (DASH) in three studies; and the Constant-Murley score in five studies.

### Quality assessment

The average CMS score was 63, indicating that the overall quality of the included studies was fair. Table 2 provides the actual values of the CMS. Inter-rater reliability was calculated between the mean values of CMS calculated by two authors (DP and RH). Cohen’s kappa coefficient (*k*) was 0.779661, indicating substantial agreement for the first round of methodological quality assessment. The intra-rater reliability was *k* = 0.864111 and 0.915309 for DP and RH respectively, indicating almost perfect agreement.

### Complications

The included studies reported transient complications such as flushing, local depigmentation of the skin, loss of sensory and motor control in the affected arm, loss of sleep, nausea, dizziness,^31^^,^^35^^,^^36^^,^^40^^,^^50^^,^^55^ hypotensive syncope^37^ and after-injection pain.^9^^,^^24^^,^^26^^,^^34^^,^^36^ In one patient, hydrodilatation was abandoned from unbearable pain during the procedure.^28^ Two studies reported one patient each with a glenohumeral joint infection.^44^^,^^50^

### Meta-analysis of the studies evaluating the effect of capsular distension versus corticosteroid alone

**Fig. 2:**
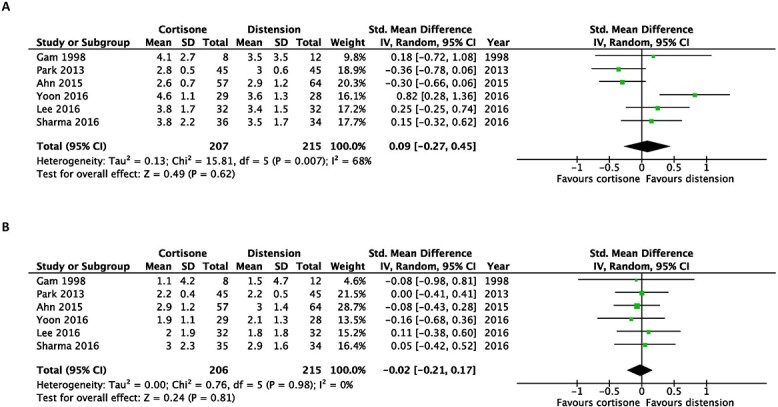
Early after intervention (**A**) and at the end of the study (**B**). Forest plot of the standardized mean differences of pain improvement comparing hydrodilatation and intra-articular corticosteroid injection.

There was no significant benefit of intra-articular corticosteroid injection alone compared with capsular distension at the first follow-up post-intervention (SMD, 0.09; 95% CI, −0.27 to 0.45) and at the last follow-up post-intervention (SMD, −0.02; 95% CI, −0.21 to 0.17) when pain scores were evaluated (Fig. 2).

**Fig. 3:**
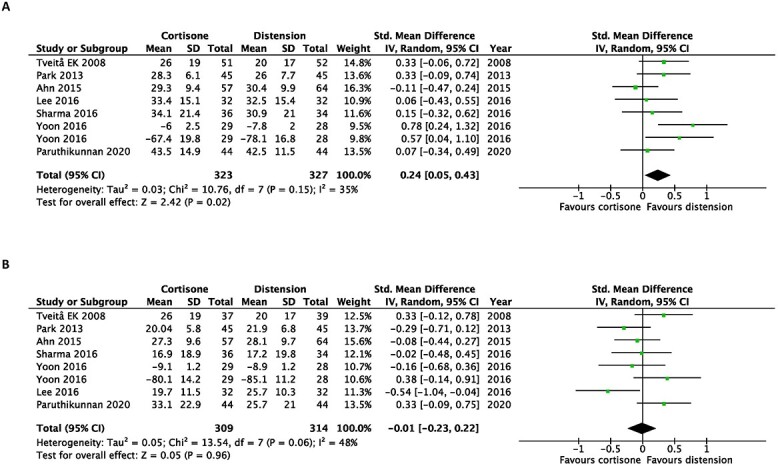
Early after intervention (**A**) and at the end of the study (**B**). Forest plot of the standardized mean differences of disability improvement comparing hydrodilatation and intra-articular corticosteroid injection.

In terms of disability, hydrodilatation was favoured over intra-articular corticosteroid injection at first follow-up post-intervention (SMD, 0.24; 95% CI, 0.05–0.43). However, this was not observed at the last follow-up post-intervention (SMD, −0.01; 95% CI, −0.23 to 0.22) (Fig. 3).

**Fig. 4:**
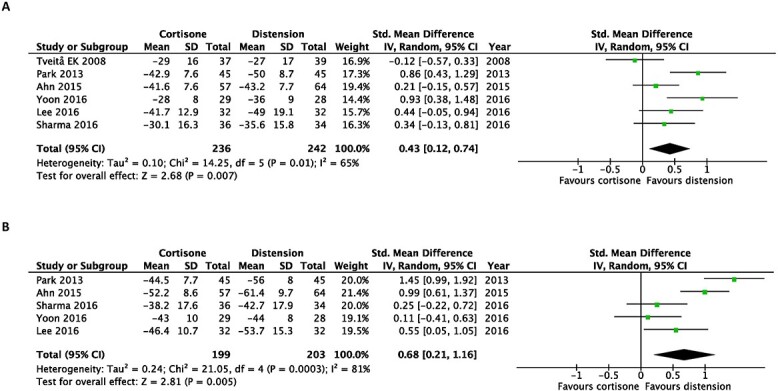
Early after intervention (**A**) and at the end of the study (**B**). Forest plot of the standardized mean differences of improvements in passive external rotation with the use of hydrodilatation or an intra-articular corticosteroid injection.

Regarding improvements in passive shoulder ROM, hydrodilatation prevailed over intra-articular corticosteroid injections when assessing passive external rotation at the earliest (SMD, 0.43; 95% CI 0.12–0.74) and at the latest follow-up post-intervention (SMD, 0.68; 95% CI, 0.21–1.16) (Fig. 4).

**Fig. 5:**
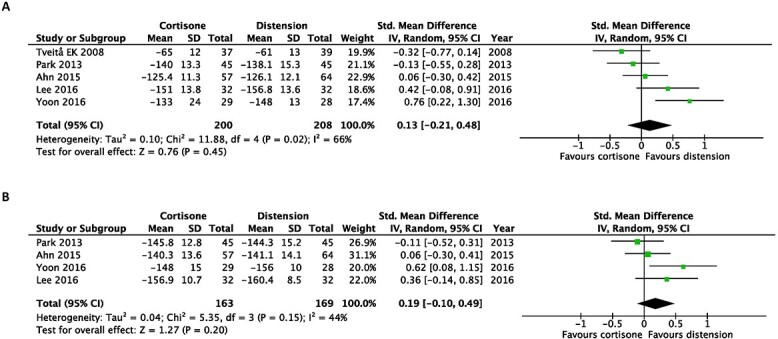
Early after intervention (**A**) and at the end of the study (**B**). Forest plot of standardized mean differences of improvements in passive forward flexion with the use of hydrodilatation or an intra-articular corticosteroid injection.

Moreover, there were no statistically significant differences in passive forward flexion, abduction or internal rotation at both time points (Figs 5–Fig. 7).

**Fig. 6:**
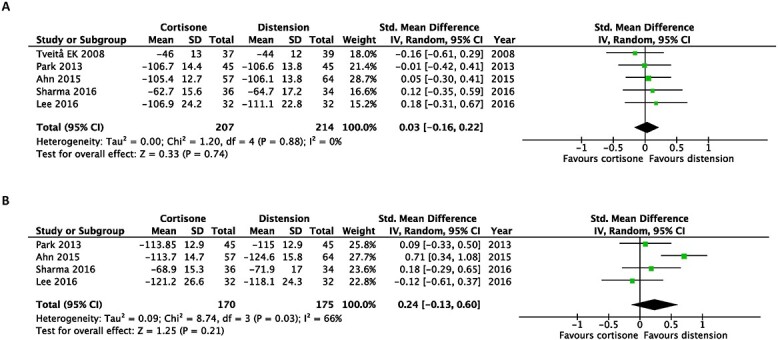
Early after intervention (**A**) and at the end of the study (**B**). Forest plot of standardized mean differences of improvements in passive abduction with the use of hydrodilatation or an intra-articular corticosteroid injection.

**Fig. 7:**
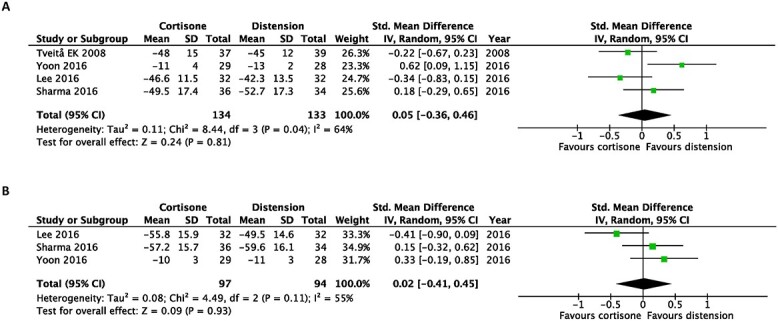
Early after intervention (**A**) and at the end of the study (**B**). Forest plot of the standardized mean differences of improvements in passive internal rotation with the use of hydrodilatation or an intra-articular corticosteroid injection.

The Cochrane Handbook Chapter 10 advises that tests for funnel plot asymmetry should only be used if a minimum of 10 studies are included in the meta-analysis. As this threshold was not reached, funnel plot asymmetry was not calculated.^17^

### Quantitative analysis of the studies not included in the meta-analysis

**Fig. 8:**
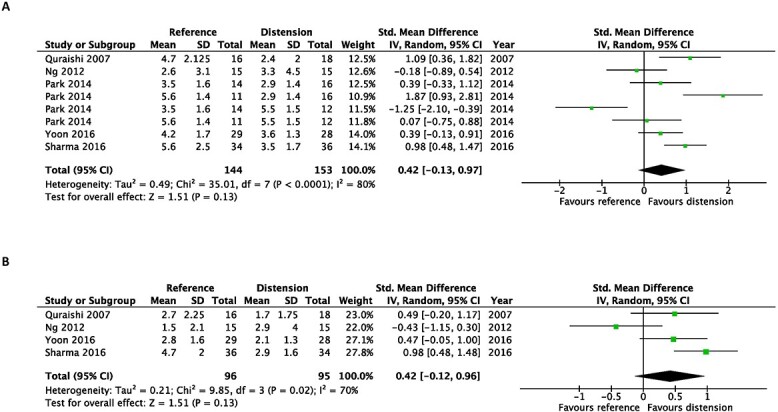
Early after the intervention (**A**) and at the end of the study (**B**). Forest plots of the standardized mean differences of improvements in pain with usage of hydrodilatation and/or different reference treatments.

**Fig. 9:**
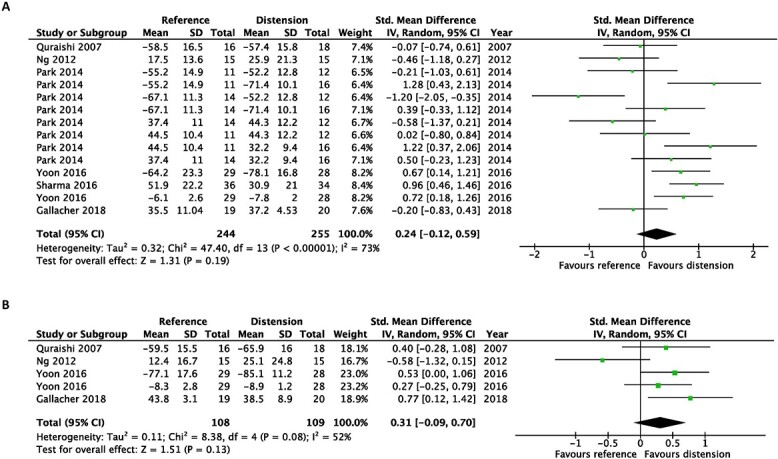
Early after the intervention (**A**) and at the end of the study (**B**). Forest plots of the standardized mean differences of improvements in disability with usage of hydrodilatation and/or different reference treatments.

**Fig. 10:**
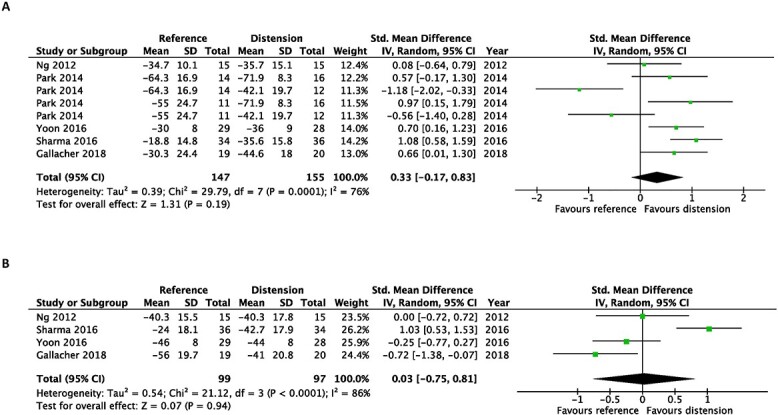
Early after the intervention (**A**) and at the end of the study (**B**). Forest plots of standardized mean differences of improvements in external rotation with usage of hydrodilatation and/or different reference treatments.

The pooled effect sizes of studies not included in the meta-analysis where intra-articular corticosteroid was not used as a control are shown in forest plots (Figs 8–10). All comparisons were not statistically significant when evaluating the pooled effect size.

Park et al.^42^ showed large effect sizes at the outcome measurements for pain, disability and external rotation for the earliest follow-ups post-intervention. In that study, a combination of intensive mobilization after hydrodilatation was compared with general physiotherapy.

## Discussion

The present systematic review investigated the effectiveness of hydrodilatation for frozen shoulder in terms of pain, shoulder disability and ROM, which were considered proxy indicators of therapeutic effects. Hydrodilatation demonstrated transient improvements in shoulder disability during the early follow-up periods. Additionally, significant improvements in passive external rotation were observed at the earliest and latest follow-ups. When comparing the pooled effects of hydrodilatation to other reference treatments, such as MUA, ACR and general physiotherapy, no significant differences were identified.

Contracture of the coracohumeral ligament is considered the predominant pathology in frozen shoulder. During image-guided hydrodilatation, leakage of contrast agents into the subscapularis bursa is often a sign of capsular rupture.^11^ This occurrence suggests that, in comparison with the posterior capsule, the anterior joint capsule is less resistant to the stretching forces of the injectate, which may account for the improvements in passive external rotation. However, more research is required to confirm this hypothesis. Various epidemiological studies have identified a link between diabetes mellitus and frozen shoulder.^57–59^ Indeed, this systematic review included a total of 224 individuals (18%) with diabetes mellitus.

In a previous Cochrane review, Buchbinder et al. identified one study comparing hydrodilatation versus placebo, and found improvements in shoulder pain and ROM. However, there was insufficient evidence to suggest that hydrodilatation prevailed over intra-articular corticosteroid injections, which are well reported for the treatment of a frozen shoulder.^10^ The combination of the two treatments may induce a synergistic effect, the former abating glenohumeral joint inflammation and the latter facilitating joint cavity expansion.^11^

Most of the evidence in the present systematic review is derived from comparisons between hydrodilatation versus intra-articular corticosteroid injections alone. The results of this review support previous studies, which also found statistically significant but transient improvements in shoulder disability and passive external rotation.^11^ Thus, clinicians must balance the immediate improvements in disability and external rotation with the possible negative consequences of hydrodilatation, such as the acute pain following joint capsular rupture. However, we did also identify improvements in passive external rotation at the latest follow-ups, contrary to the findings of previous studies.^11^

Furthermore, mixed results were evidenced when comparing the efficacy of hydrodilatation and MUA. Park et al. found statistically significant improvements in pain, disability and external rotation for MUA when compared with hydrodilatation.^42^ On the other hand, Quraishi et al. identified that hydrodilatation provided statistically significant improvements in pain compared with MUA in the earliest follow-up periods.^51^ However, there were no significant differences in pain scores at late follow-ups and in terms of disability outcome measures. Therefore, MUA should be considered secondary to hydrodilatation given its uncertainty regarding its superiority. Also, MUA is a relatively expensive inpatient procedure, whereas hydrodilatation is an outpatient treatment which does not require anaesthesia. Other recognized drawbacks of MUA include humeral fractures, isolated infraspinatus paralysis, brachial plexus traction injuries and rotator cuff tears.^47^^,^^49^^,^^51^

## Limitations

This investigation presents several limitations. Firstly, as frozen shoulder of all durations was examined as a whole, we could not determine the best way to treat each of the stages of frozen shoulder. Secondly, both within and across trials, different volumes of hydrodilatation fluid were utilized. As a result, we were unable to assess the association between injectate volume and its clinical efficacy. Therefore, to standardize the delivery of hydrodilatation in future studies, researchers and clinicians should adhere to recently published guidelines.^60^ Thirdly, our secondary outcomes included several shoulder ROM components that might lead to erroneous positive results. As a result, any favourable secondary outcomes should be carefully assessed and supported by further research. Fifthly, publication bias was not assessed, as we had less than ten studies in the meta-analysis. Sixthly, our meta-analysis software (Review Manager 5.4) was not able to differentiate the specific outcomes measures and comparative treatments on the forest plots for the studies by Park (2014) and Yoon (2016) (Figs 8–10). This made it impossible to visually distinguish which comparative treatment demonstrated superior efficacy.

Furthermore, only a relatively few outcomes, namely changes in pain intensity, disability and improvements in ROM, were used to assess the efficacy of hydrodilatation. As a result, several potentially important outcomes were not considered, including patient satisfaction and incidence of complications. Also, the role of concurrent physiotherapy on the effects of hydrodilatation was not measured since patients’ post-intervention exercise routines differed among the included trials and were not described in sufficient detail. Therefore, future research should include standardized rehabilitation protocols, and ensure that the regimen is adequately described.^61^ Finally, doubts regarding the accuracy of injections should be considered as we did not differentiate the study’s results based on image- versus anatomical landmark-guided injections.

## Conclusion

Hydrodilatation may provide early improvements of disability in addition to short- and long-term improvements in passive external rotation in frozen shoulder. However, there is comparable effectiveness of glenohumeral joint hydrodilatation and intra-articular corticosteroid injection when assessing most long-term outcomes. Hydrodilatation is a promising alternative treatment to the more expensive surgery. Clinicians must manage patient expectations appropriately given the wide number of reported complications. Finally, well-designed, appropriately powered RCTs, with a low risk of bias, are required to confirm the relevance and validity of hydrodilatation in the management of frozen shoulder.

## Data Availability

All the data underlying the submission in the manuscript has been reported.

## CRediT author statement

Daryl Poku (Conceptualization, Data curation, Formal analysis, Investigation, Methodology, Writing—original draft), Rifat Hassan (Conceptualization, Data curation, Formal analysis, Investigation, Methodology, Writing—original draft), Filippo Migliorini (Methodology, Software, Validation, Visualization), Nicola Maffulli (Methodology, Software, Validation, Visualization, Writing—review and editing).
